# Renal calcified mass misdiagnosed as a renal calculus in an adult with tuberculosis “autonephrectomy”: a case report

**DOI:** 10.4076/1757-1626-2-7613

**Published:** 2009-08-06

**Authors:** Amie Victoria Clifford, Aidan Paul Noon, Daniel Raw, James Hall

**Affiliations:** 1 The Medical School, University of SheffieldBeech Hill Road, Sheffield, S10 2RXUK; 2 The Department of Urology, Royal Hallamshire HospitalGlossop Road, Sheffield, S10 2JFUK; 3 The Department of Radiology, Barnsley District GeneralGawber Road, Barnsley, South Yorkshire, S75 2EPUK; 4 The Department of Urology, Royal Hallamshire HospitalGlossop Road, Sheffield, S10 2JFUK

## Abstract

**Introduction:**

Tuberculosis was once a disease much more prominent in the minds of UK urologists. The dramatic reduction in incidence following the success of antituberculous therapy has meant that new generation surgeons have little or no experience of the effects and management of tuberculosis of the kidney. With concern over multidrug resistant tuberculosis, human immunodeficiency virus associated tuberculosis and immigration of persons from areas endemic with this disorder, clinicians may see an increase in cases of renal tuberculosis. Renal tuberculosis “autonephrectomy” is the end stage of chronic renal tuberculosis infection and results from the caseous necrosis and progressive cavitation of the kidney. Resultant calcification may mimic the appearances of a renal calculus on plane film X-ray. Back, flank and abdominal pain are non-specific symptoms often investigated by General Practitioners using plane film X-ray. Clinicians not considering a diagnosis of renal tuberculosis may confuse the radiographic appearances with that of a renal calculus as occurred in our case. Once a diagnosis of tuberculosis autonephrectomy is made the next decision is whether any further investigations and treatment is necessary as the condition has been reported to be a cause of hypertension and reactivation of tuberculosis is also possible.

**Case presentation:**

We describe the case of a 66 year old Caucasian female who presented to her General Practitioner with left sided lumber and loin pain. A lumbar spine X-ray showed a calcified mass reported as a renal calculus. Urological opinion was sort and a computerised tomogram confirmed a renal tuberculosis “auto nephrectomy”. The patient had been diagnosed with tuberculosis aged 16. The patient had no lower urinary tract symptoms and normal urinalysis. Although there is some evidence to suggest nephrectomy is beneficial in treating hypertension in these patients (the patient in our case was on two anti hypertensive preparations), the patient did not want to consider surgery as her symptoms had settled spontaneously.

**Conclusion:**

Although very rare in non endemic countries clinicians still need to consider a diagnosis of renal tuberculosis in patients with previous tuberculosis exposure and calcification of the urinary tract. In cases of uncontrolled hypertension consideration should be given to nephrectomy in cases of end stage renal tuberculosis. This decision should be made in consultation with a nephrologist.

## Introduction

The estimated tuberculosis (TB) incidence in the UK for 2007 was 15 per 100 000 population with a prevalence of 12 per 100 000 population [1]. Genito-urinary TB accounts for 15-20% of cases of extrapulmonary TB [2], of these about 25% will have had known pulmonary TB at some stage. Symptoms typically include dysuria, frequency and haematuria, which may be macroscopic or microscopic. Back, flank and abdominal pain may also be present. However there may be a long latency period before clinical manifestation of renal tuberculosis occurs. Following pulmonary primary infection there is a haematogenous phase where the glomerular and peritubular capillary bed of the kidneys become seeded with *M tuberculosis* [2,3]. The bacilli may then proliferate in this region and the resulting cortical granulomas may remain dormant for many years. However a break down of host immunity may result in reactivation of these granulomas, which then enlarge, coalesce and as a result of capillary rupture then spread to the proximal tubule and loop of Henle. Through this process of granuloma formation, caseous necrosis and cavitation the entire kidney can be destroyed leading to “auto-nephrectomy” (as in our case) [2,3].

Diagnosis of acute renal TB rests on three first-morning-void urine samples. An acid-fast stain and mycobacterial culture are then performed. The results may also be supported by imaging findings. A plain abdominal X-ray may show enlargement of a kidney and calcification of the kidneys and lower urinary tract [3]. Computed tomography (CT) may help to detect renal TB and any extra-renal spread. It is also the most sensitive method of identifying renal calcification [4]. However ultrasound can also reveal calcifications, renal contractions and scars. Based on the imaging appearance of renal TB the differential diagnosis includes chronic pyelonephritis, papillary necrosis, medullary sponge kidney, caliceal diverticulum, renal cell carcinoma, transitional cell carcinoma and xanthogranulomatous pyelonephritis [2].

The management of acute renal TB requires referral to a TB expert and in many cases where the kidneys are functioning, adequately draining, and having only small cavities and scars - an intensive course of antituberculous drug therapy will be sufficient to sterilize the renal lesion and will not require the attention of a urologist [2].

## Case presentation

**Figure 1.**
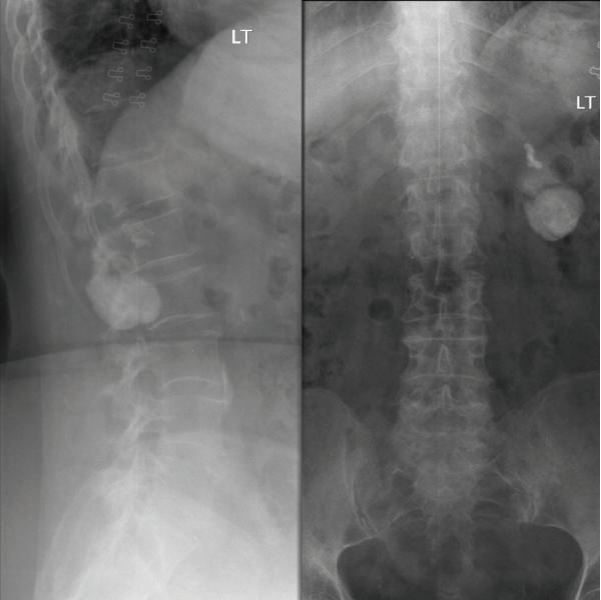
AP & Lateral lumbar spine X-rays.

A 68-year-old Caucasian lady presented with left sided upper abdominal pain and left renal angle pain to her GP. The GP ordered lumbar X-rays of the spine, which had shown a possible 7 cm renal stone (Figure 1). The pain had been occurring for several months and there were no exacerbating or relieving factors. The pain did not radiate and was not severe. She had no lower urinary tract symptoms importantly no haematuria or dysuria. Other medical problems included rheumatoid arthritis, SLE and controlled hypertension. She had no previous urological or surgical history. She was referred to a urologist for an opinion.

**Figure 2.**
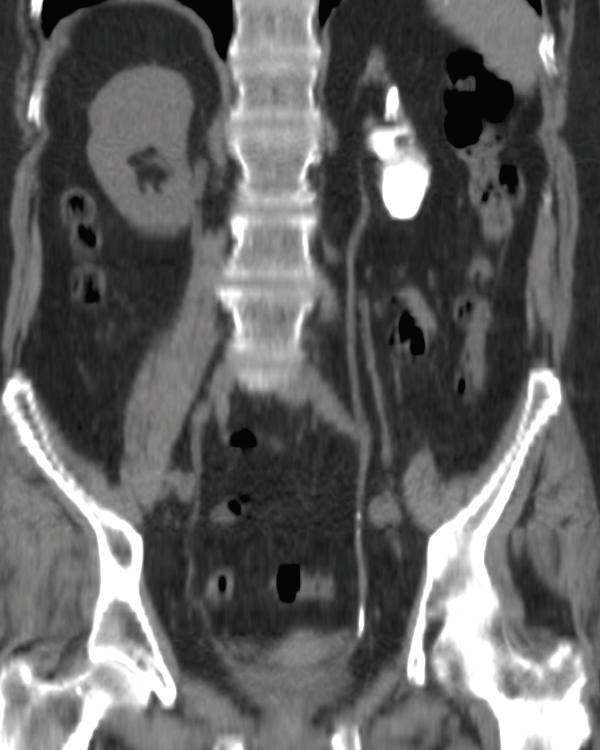
Non-contrast CT, coronal curves reformat.

Laboratory investigations showed a serum creatinine of 119 mg/dl and a urea of 8.1 mg/dl. Urinalysis was normal and her blood pressure was 140/80 mmHg. Her symptoms of back pain had subsided and on examination she had no loin pain. She was subsequently sent for a CT scan, which revealed that the substance of her left kidney had almost totally been replaced by a dense calcification. There was also calcification of the distal ureteric wall on the same side. Both of these findings are evident from the reconstructed CT image (Figure 2). When she was subsequently reviewed again in clinic, it came to light that she had had TB in her chest at the age of 16. A diagnosis of TB “auto nephrectomy” was made. From the time of her original referral she had no further episodes of pain and it was felt that her symptoms had been due to musculoskeletal problems and not due to the TB kidney. A review of the present literature suggested that nephrectomy may be considered for patients that are hypertensive. Our patient had well controlled hypertension and after discussion with the patient it was not felt that nephrectomy was warranted. She was discharged back to her GP, with advice to monitor her blood pressure and renal function.

## Discussion

What to do surgically with TB autonephrectomy is controversial. One school of thought is that a 2-year course of medical treatment will sterilize the end stage kidney [2], possibly preventing the risk of reactivation. Butler and O’Flynn [5] in a series of 838 cases of genitourinary tuberculosis reported reactivation of genitourinary tuberculosis in only 53 of these cases (6%) and recommended life-long follow up. However others authors have proposed that a secondary nephrectomy should be performed in order to prevent reactivation and shorten the duration of medical therapy [2].

Carl and Stark [6] recommended that if the kidney is completely destroyed by caseous decay a secondary nephrectomy is unavoidable however this is a debatable point if the kidney is non functioning and symptomless. It has been demonstrated that hypertensive patients with unilateral tuberculous nephropathy may benefit in particular from a nephrectomy [5]. In a study by Flechner and Gow [7] 17 hypertensive patients with non-functioning or poorly functioning kidneys underwent a nephrectomy which resulted in a significant reduction in blood pressure (at least a 25 mmHg systolic and 10 mmHg diastolic decrease) for 11 of these patients (64.7%). Nine of these patients were followed up for at least 3 years, none of which deteriorated or were on antihypertensives.

With the development of antituberculous drugs nephrectomy has become less important. However a nephrectomy may still be valuable in the following circumstances: (i) if the kidney is calcareous, destroyed and subsequently causing pain or an abscess; (ii) the patient has hypertension alongside a unilateral renal lesion; (iii) recurrent UTI’s causing persistent symptoms; (iv) suspicion of malignancy in one of the kidneys which has been damaged by renal TB. Furthermore they are the cause of (i) abscess formation with cutaneous sinus tracts and (ii) systemic hypertension which are the complications that may occur in the end stage non- functioning kidney. It is unlikely that chemotherapy alone prevents these complications from occurring as both are associated with sterile organs [5].

A series by Flechner and Gow [7] reviewed 300 cases of genitourinary tuberculosis. 73 of these patients had a standard lumbar nephrectomy following 6 weeks of antituberculous chemotherapy because an excretory urogram had shown them all to have unilateral non-functioning or poorly functioning kidneys. There was no perioperative mortality. All the patients received post-operative observation and follow up for a mean time of 9.4 years and urine samples did not yield mycobacteria. There were four patients who had a unilateral non-functioning kidney and did not have a primary nephrectomy. Three of these four patients subsequently had delayed complications however the fourth was lost to follow up.

## Conclusion

Although very rare in non endemic countries clinicians still need to consider a diagnosis of renal TB in patients with previous TB exposure and calcification of the urinary tract. For patients with suspected active disease they should be seen by an expert in TB management. Where there is evidence that the disease is end stage such as “autonephrectomy” often no treatment is needed. In cases of uncontrolled hypertension consideration should be given to nephrectomy, similarly uncontrolled pain associated with the kidney may be an indication for nephrectomy. This decision should be made in consultation with a nephrologist. Patients that are immunocompromised should be referred to a TB expert for advice on whether reactivation is a risk.

## Abbreviations

CT: computerised tomogram
SLE: systemic lupus erythematous
TB: tuberculosis
UTI: urinary tract infection
